# Optimal group sizes for testing group mean differences using the Bayes factor

**DOI:** 10.1080/02664763.2025.2534898

**Published:** 2025-07-24

**Authors:** Mirjam Moerbeek

**Affiliations:** Department of Methodology and Statistics, Utrecht University, Utrecht, The Netherlands

**Keywords:** Analysis of variance, Bayes factor, informative hypothesis, optimal design methodology, budgetary constraint

## Abstract

Determination of group sizes is an important issue when planning a study that aims to compare mean outcomes across groups. Using equal group sizes is not the best choice in the case of heterogeneous costs and/or variances. Conventional optimal design methodology has shown that groups with higher variance and lower costs should include more subjects. However, these results are based on the framework of null hypothesis significance testing, which has received severe criticism over the past decades. The Bayesian approach to hypothesis testing has been proposed as an alternative and uses the Bayes factor to quantify the support of a hypothesis given the data. Group sizes that maximize the Bayes factor are determined, and it is shown how these optimal group sizes depend on the variances, costs and group means. Furthermore, it is shown to what degree the Bayes factor becomes smaller while using conventional optimal design methodology or equal group sizes. The optimal design methodology is illustrated using examples on multidisciplinary pain management and psychological status and asthma outcomes. A Shiny app has been made available to facilitate the use of the optimal design methodology.

## Introduction

Analysis of variance is a statistical technique for the comparison of group means on a continuous outcome variable. An important question in the design phase of a study is how large each of the groups should be. Often, a balanced design with equal group sizes is chosen. A rationale for such a design in clinical trials is that it is consistent with the view of clinical equipoise that must exist before the start of a trial [47]. However, the balanced design is not necessarily the most efficient choice, especially so when variances and/or costs are heterogenous across groups [43,48,45]. To achieve a more efficient design, the groups with the highest variance and/or lowest costs should consist of more subjects than those with the lowest variance and/or highest costs.

Over the past twenty years, various papers on optimal group sizes in analysis of variance appeared [28,29,15,14,16,1]. Special attention has been paid to placebo-treatment comparisons [53,7,42,54,52], cluster randomized trials [27,31,23,26,24] and multicenter trials [25]. The research question in these papers is: given a fixed total sample size or a fixed budget to include subjects in the study, what should be the optimal allocation of subjects to the groups?

These papers have in common that they focus on the framework of null hypothesis significance testing [10], where the *p*-value is used to decide if the null hypothesis $H_{0}:\mu_{1}=\mu_{2}=\ldots =\mu_{K}$ of equal means μ across the K groups should be rejected or not. Over the past decades, this approach to hypothesis testing has received severe criticism in numerous papers [44,37,49,5,30,51]. One of the main points of criticism is that in many studies, one is not really interested in the null hypothesis. One may have prior expectations, based on expert knowledge or the literature, that the one group has a higher mean than another. For instance, consider a hypothetical study on the comparison of two pain killers A and B to a control C. If one expects pain killer A to result in lower pain levels than pain killer B and the control having the highest pain levels, then one may formulate the following so-called informative hypothesis: $H:\mu_{A}<\mu_{B}<\mu_{C}$, where lower mean scores μ imply lower pain levels. Testing such a hypothesis in the framework of null hypothesis significance testing is burdensome. One approach is to test the traditional null hypothesis $H_{0}:\mu_{A}=\mu_{B}=\mu_{c}$. If the *p*-value is sufficiently small, one rejects the null hypothesis but does not yet know where differences among treatment means can be found. Such differences may be found in a post-hoc test based on pairwise comparisons, correcting for multiple hypothesis testing. Another approach is to formulate a set of a priori contrasts: $H_{01}:\mu_{A}=\mu_{B}$ and $H_{02}:\mu_{B}=\mu_{C}$. The corresponding one-sided alternative hypotheses reflect the order of the treatment means in the informative hypothesis above: $H_{A1}:\mu_{A}<\mu_{B}$ and $H_{A2}:\mu_{B}<\mu_{C}$. Again, correcting for multiple hypothesis testing should be done. The drawback of both approaches, however, is that they do not test the informative hypothesis directly.

Another drawback of null hypothesis significance testing is that the *p*-value provides evidence *against* the null hypothesis, while one would prefer a measure *in favor* of a hypothesis, whenever it is a null or informative hypothesis. Such a measure is the Bayes factor, which was introduced already a few decades ago [20,19] and has been further developed over the past twenty years [13,18,22]. The Bayes factor is a measure of support or evidence for a specific hypothesis relative to another hypothesis, given the data. It can thus be used to compare competing hypotheses to each other. For instance, if $BF_{12}=5$, then there is five times more support in the data for hypothesis $H_{1}$ than for a competing hypothesis $H_{2}$.

Recently, a few papers on a priori sample size determination when testing hypotheses on group means using the Bayes factor have been published [8,9]. In these papers, the research question is: how large should the size of the groups be so that the Bayes factor exceeds a user-selected threshold with a certain probability? Attention has also been paid to Bayesian sequential designs, where additional data are collected until the Bayes factor exceeds a user-selected threshold [41,33,32,46]. It should be noted that these papers have in common that they focus on equal group sizes.

The approach to sample size determination in this contribution is different from that in the references above. Here, we start with either a fixed total number of subjects or a fixed budget for including subjects in the study and derive the optimal size for each of the groups. So, we do not focus on equal group sizes since it may be very likely that assigning more subjects to groups with higher variance and/or lower costs and fewer subjects to groups with lower variance and/or higher costs results in a higher Bayes factor. Furthermore, it may be expected that the differences in group means also influence the optimal group sizes. For instance, if the difference in group means between pain killers A and B is smaller than the difference between pain killers B and C, then it makes sense to assign more subjects to A and B and fewer to C. Once the optimal group sizes have been derived, the Bayes factor that can then be achieved is calculated.

To my knowledge, no research has been done on optimal group sizes for hypothesis testing using the Bayes factor. This paper develops a methodology to fill this gap. The next section reviews findings on optimal group sizes from the perspective of null hypothesis significance testing. The following section gives a short introduction to the Bayes factor and develops methodology to derive optimal group sizes for the Bayesian approach to hypothesis testing. The focus is on studies where either the total sample size or the budget to include subjects in the study is fixed beforehand. Subsequently, it is evaluated how optimal group sizes are influenced by the group-specific costs to include a subject, the within-group variances and the differences in group means. The Bayes factor achieved with the optimal group sizes is compared to the Bayes factor achieved with equal group sizes and to the Bayes factor achieved with optimal group sizes based on null hypothesis significance testing. The methodology is illustrated using a study on pain management [4] and another study on psychological status and asthma outcomes [11] as examples. The paper concludes with a discussion and directions for future research.

## Optimal design methodology

The continuous outcome of subject $j=1,\ldots ,n_{k}$ within group k=1,…,K follows a normal distribution:

(1) $$y_{\mathrm{jk}}∼N(\mu_{k},\sigma_{k}^{2}).$$

Not only the group means $\mu_{k}$ but also the within-group variances $\sigma_{k}^{2}$ may vary across the groups. The group means in the vector $\mu ={\left(\begin{array}{cccc} \mu_{1} & \mu_{2} & \ldots & \mu_{K} \end{array}\right)}^{\prime }$ are estimated by the average scores within each group: $\hat{\mu }={\left(\begin{array}{cccc} {\bar{y}}_{.1} & {\bar{y}}_{.2} & \ldots & {\bar{y}}_{.K} \end{array}\right)}^{\prime }$ with the associated covariance matrix

(2) $$\hat{\mathrm{cov}}(\hat{\mu })=\left(\begin{array}{cccc} \frac{{\hat{\sigma }}_{1}^{2}}{n_{1}} & 0 & \cdots & 0\\ 0 & \frac{{\hat{\sigma }}_{2}^{2}}{n_{2}} & \cdots & 0\\ \vdots & \vdots & \ddots & \vdots \\ 0 & 0 & \cdots & \frac{{\hat{\sigma }}_{K}^{2}}{n_{K}} \end{array}\right),$$

where ${\hat{\sigma }}_{k}^{2}$ is the variance estimate in group k. As the groups are independent, the covariances on the off-diagonal are equal to zero.

A design $\xi =(n_{1},n_{2},\ldots ,n_{K})$ is a combination of positive integer group sizes in the design space Ω. The design space is determined by a budgetary constraint: the costs for recruitment, incentives, implementation of treatment (in the case of a trial) and measurement do not exceed the budget that is available. Given costs $c_{k}$ per subject within group k, and budget B the budgetary constraint is

(3) $$\overset{K}{\underset{k=1}{\sum }}c_{k}n_{k}\leq B.$$

A special case is $c_{k}=1,\forall k$ and B=N, where N is the total sample size. In that case, the optimal design is sought under a fixed total sample size rather than a fixed budget. Furthermore: $n_{k}\geq n_{k.\text{min}}$. Minimum group sizes $n_{k.\text{min}}=2$ are needed to be able to estimate the within-group variances. However, larger group sizes are often used in practice to get less biased estimates.

In the case of equal group sizes

(4) $$n_{k}=n=\frac{B}{\sum_{k=1}^{K}c_{k}},\mathrm{\forall k},$$

which is rounded downwards in the case of a non-integer value.

We use optimal design methodology [2,3] to determine the optimal design $\xi^{*}=(n_{1}^{*},n_{2}^{*},\ldots ,n_{K}^{*})$. The optimal design minimizes an optimality criterion Φ.

We first focus on a criterion that follows from testing the null hypothesis $H_{0}:\mu_{1}=\mu_{2}=\ldots =\mu_{K}$. In the case of heterogenous within-group variances, the null hypothesis is tested using Welch’s F test [50]. The non-centrality of the F test statistic is

(5) $$\lambda =\frac{K\sum_{k=1}^{K}{\left(\hat{\mu }-{\hat{\mu }}_{k}\right)}^{2}}{\sum_{k=1}^{K}\frac{{\hat{\sigma }}_{k}^{2}}{n_{k}}},$$

where $\hat{\mu }$ is the estimated grand mean. The statistical power to reject the null hypothesis is maximized when the non-centrality parameter is maximized, that is, when its denominator is minimized. The optimality criterion is thus

(6) $$\Phi_{A}=\min\overset{K}{\underset{k=1}{\sum }}\frac{\sigma_{k}^{2}}{n_{k}},$$

where an a priori estimate of the variance $\sigma_{k}^{2}$ is used.

This optimality criterion is known as the A-optimality criterion and minimizes the sum of the diagonal elements of $\mathrm{cov}(\hat{\mu })$. The optimal design is achieved when using the optimal group size allocation ratio:

(7) $$\frac{n_{k}}{n_{1}}=\sqrt{\frac{\sigma_{k}^{2}}{\sigma_{1}^{2}}\frac{c_{1}}{c_{k}}},$$

See [16]. This only results in equal group sizes when the argument of the square root is equal to 1. This condition holds when either $c_{k}=c,\mathrm{\forall k}$ and $\sigma_{k}^{2}=\sigma^{2},\forall k$ or $\frac{\sigma_{k}^{2}}{\sigma_{1}^{2}}=\frac{c_{k}}{c_{1}}$, ∀k.

The second optimality criterion follows from estimating the group means with maximum efficiency. The D-optimal design minimizes the determinant of $\mathrm{cov}(\hat{\mu })$, which is the product of its diagonal elements:

(8) $$\Phi_{D}=\min\overset{K}{\underset{k=1}{\prod }}\frac{\sigma_{k}^{2}}{n_{k}}.$$

In other words, the D-optimal design minimizes the k-dimensional confidence ellipsoid of the vector $\hat{\mu }$. The function to be minimized is non-linear with respect to its arguments $n_{1}$, $n_{2}$, … , $n_{K}$ and is to be minimized subject to the budgetary constraint $\sum_{k=1}^{K}c_{k}n_{k}\leq B$ and subject to $n_{k}\geq n_{k.\text{min}}$. A solution to this non-linear optimization problem may be found using the R package alabama [40] and rounding off to integer values. Alternatively, one may evaluate the values of the determinant of $\mathrm{cov}(\hat{\mu })$ for all possible designs $\xi =(n_{1},n_{2},\ldots ,n_{K})$ in the design space Ω and select the design for which this determinant is minimized.

## Testing hypotheses using the Bayes factor

This section gives a short introduction to hypothesis testing using the Bayes factor. For a more extensive tutorial, the reader is referred to ref. [18]. In this introduction, three groups are used; the extension to more than three groups is straightforward. Consider a study with three groups and the informative hypothesis $H_{1}:\mu_{1}<\mu_{2}<\mu_{3}$, with lower group means μ indicating better results. This hypothesis is to be compared to its complement $H_{1c}$, which consists of all 3!−1=5 other possible orderings of group means ( $H_{2}:\mu_{1}<\mu_{3}<\mu_{2}$, $H_{3}:\mu_{2}<\mu_{1}<\mu_{3}$, $H_{4}:\mu_{2}<\mu_{3}<\mu_{1}$, $H_{5}:\mu_{3}<\mu_{1}<\mu_{2}$, $H_{6}:\mu_{3}<\mu_{2}<\mu_{1}$). The corresponding Bayes factor is calculated as

(9) $$BF_{1C}=\frac{f_{1}/c_{1}}{f_{c}/c_{c}}.$$

Here, $c_{1}$ is the complexity of hypothesis $H_{1}$ and is defined as the proportion of the prior distribution that is in agreement with $H_{1}$. For equal within-group variances, it can be shown $c_{1}=\frac{1}{K!}=\frac{1}{3!}=\frac{1}{6}$ [17]. In other words, the complexity is determined by the number of orderings of the K=3 group means. The complexity of the complement hypothesis $H_{1c}$ is simply $1-c_{1}$. Furthermore, $f_{1}$ is the fit of the hypothesis $H_{1}$ and is defined as the proportion of the posterior distribution that is in agreement with $H_{1}$. The fit of the complement hypothesis $H_{1c}$ is simply $1-f_{1}$.

In this paper, the Approximated Adjusted Fractional Bayes Factor (AAFBF) is used [38,13]. A fraction of the data is used to specify the prior distribution of the group mean vector $\mu ={\left(\begin{array}{ccc} \mu_{1} & \mu_{2} & \mu_{3} \end{array}\right)}^{\prime }$. This implies that the user does not need to specify the distributional form of the prior. When the hypothesis to be tested only includes inequality constraints (specified by using ‘<’ or ‘>’), then the Bayes factor does not depend on the fraction [18]. The AAFBF approach may then be considered more attractive than selecting a default prior, such as the Jeffreys prior or g prior, and specifying tuning parameters of such a prior.

The prior distribution of μ is given by

(10) $$h(\mu |y_{1})=N\left(\left(\begin{array}{c} 0\\ 0\\ 0 \end{array}\right),\left(\begin{array}{ccc} \frac{{\hat{\sigma }}_{1}^{2}}{b_{1}n_{1}} & 0 & 0\\ 0 & \frac{{\hat{\sigma }}_{2}^{2}}{b_{2}n_{2}} & 0\\ 0 & 0 & \frac{{\hat{\sigma }}_{3}^{2}}{b_{3}n_{3}} \end{array}\right)\right),$$

where $y_{1}$ are the data to construct the prior and ${\hat{\sigma }}_{k}^{2}$ is the unbiased estimate of the variance in group k, where ${\hat{\sigma }}_{k}^{2}$ is based on $y_{1}$. The prior is a tri-variate normal distribution with means equal to zero. This implies the prior distribution is not used to represent prior information about differences in group means. This information is part of the hypothesis $H_{1}:\mu_{1}<\mu_{2}<\mu_{3}$ rather than the prior. The variances depend on the fractions $b_{k},k=1,2,3$ that are taken from the data to construct the prior. The default value is $b_{k}=\frac{J}{K}\times \frac{1}{n_{k}}$, where K=3 is the number of groups and J=K−1=2 is the number of equality constraints (specified by using ‘+’) used to specify the null hypothesis $H_{0}:\mu_{1}=\mu_{2}=\mu_{3}$ on these three group means [18]. However, as was mentioned above, the value of the fraction does not influence the Bayes factor in the case of testing inequality-constrained hypotheses.

The prior $y_{2}$, which is the part of the data that is not used to construct the prior, is used to construct the posterior of μ:

(11) $$g(\mu |y_{2})=N\left(\left(\begin{array}{c} {\hat{\mu }}_{1}\\ {\hat{\mu }}_{2}\\ {\hat{\mu }}_{3} \end{array}\right),\left(\begin{array}{ccc} \frac{{\hat{\sigma }}_{1}^{2}}{n_{1}} & 0 & 0\\ 0 & \frac{{\hat{\sigma }}_{2}^{2}}{n_{2}} & 0\\ 0 & 0 & \frac{{\hat{\sigma }}_{3}^{2}}{n_{3}} \end{array}\right)\right),$$

where ${\hat{\mu }}_{k}$ is the maximum likelihood estimate of the mean in a group k, and it is based on $y_{2}$.

The R packages bain [12] and BFpack [35] can be used to calculate the AAFBF. The first simulates data from the prior and posterior to calculate fit and complexity; the latter uses existing functions for multivariate normal distributions, such as pmvnorm from the R package mvtnorm [36].

Guidelines for interpretation of Bayes factors can be found in the literature [21,19], see Table 1. It should be noted that the Bayes factor is a continuum, and the guidelines in Table 1 should not be used in a very stringent manner. At least they should not be used in a dichotomous decision rule, such as the type I error rate α is used in null hypothesis significance testing to distinguish significant and insignificant effects. It may even be argued not to use the guidelines in Table 1 at all and let the reader make his or her own judgement based on the reported value of the Bayes factor.

**Table 1.** Classification scheme for the Bayes factor $BF_{1c}$.

$BF_{1c}$	Interpretation
>100	Decisive support for $H_{1}$
30–100	Very strong support for $H_{1}$
10–30	Strong support for $H_{1}$
3–10	Substantial support for $H_{1}$
1–3	Mild support for $H_{1}$
1/3–1	Mild support for $H_{1c}$
1/10–1/3	Substantial support for $H_{1c}$
1/30–1/10	Strong support for $H_{1c}$
1/100–1/30	Very strong support for $H_{1c}$
<1/100	Decisive support for $H_{1c}$

## Optimal group sizes for an inequality-constrained hypothesis

In this section, we study the optimal group sizes for testing the inequality-constrained hypothesis $H_{1}:\mu_{1}<\mu_{2}<\mu_{3}$ versus its complement $H_{1c}$ subject to the budgetary constraint $n_{1}c_{1}+n_{2}c_{2}+n_{3}c_{3}\leq B$ and for all integer $n_{k}\geq 2,k=1,2,3$. The optimality criterion to be maximized is the Bayes factor $BF_{1C}$. Henceforward, the optimal design thus obtained will be referred to as the BF-optimal design. Unfortunately, a closed form expression for the optimal design does not exist. Therefore, the Bayes factor is calculated for all possible designs ξ in the design space Ω. The group sizes for which the Bayes factor is maximized are the optimal group sizes. Finding the optimal design in such a way is computationally intensive. To decrease calculation time, it makes sense to only evaluate those designs for which $B-c_{\text{max}}\leq n_{1}c_{1}+n_{2}c_{2}+n_{3}c_{3}\leq B$, where $c_{\text{max}}=\text{max}(c_{1},c_{2},c_{3})$. Only designs that have costs of at least $B-c_{\text{max}}$ are considered as designs that have lower costs do not efficiently use the budget. In other words, it is still possible to add at least one subject (in whichever group) to such a design and hence reach a higher Bayes factor. Furthermore, we focus on designs with costs of at most B but not necessarily equal to B. Group sizes are integers so it is not always possible to fully spend the budget. One may even further decrease calculation time by placing a maximum on one or more group sizes, for instance when one or more groups consist of subjects with a rare disease or disorder or from a small subpopulation, or when one or more of the groups receive a treatment that may have harmful side effects to which a limited number of subjects should be exposed. As a special case we may consider $c_{1}=c_{2}=c_{3}=1$ and B=N so that the optimal design is sought given a fixed total sample size and the constraint is $n_{1}+n_{2}+n_{3}=N$. Here, it is obvious the optimal design will have exactly N subjects and not fewer than that.

The optimal design methodology is demonstrated for a fictional study with costs per subject $c_{1}=c_{2}=c_{3}=100$ and budget B=30000 and with $\sigma_{1}^{2}=\sigma_{2}^{2}=\sigma_{3}^{2}=1$ and $\mu_{1}=0.2$, $\mu_{2}=0.4$ and $\mu_{3}=0.6$. In other words, the difference between $\mu_{1}$ and $\mu_{2}$ is equal to the difference between $\mu_{2}$ and $\mu_{3}$ and of small size according to Cohen’s d. The reader may use the Shiny app to verify that the A- and D-optimal designs have equal group sizes $n_{1}=n_{2}=n_{3}=100$, a total sample size of 300 and a corresponding BF=26.8. The design that results in the highest Bayes factor has group sizes $\left(n_{1},n_{2},n_{3})=(104,92,104\right)$, a total sample size of 300 and a corresponding BF=26.9, which is hardly any larger than the one obtained with equal group sizes. We observe the BF-optimal design includes more subjects in the outer groups 1 and 3 than in the middle group 2. The output of the Shiny app also lists four designs that perform almost as well as the BF-optimal design. Each of them has more subjects in the outer groups than in the middle group. We also observe symmetry: designs (103,93,104) and (104,93,103) perform equally well and so do designs (103,92,105) and (105,92,103). This will not necessarily be the case for studies with heterogenous costs and/or variances and/or when $\mu_{1}-\;\mu_{2}\neq \;\mu_{2}-\;\mu_{3}$.

**Figure 1.**
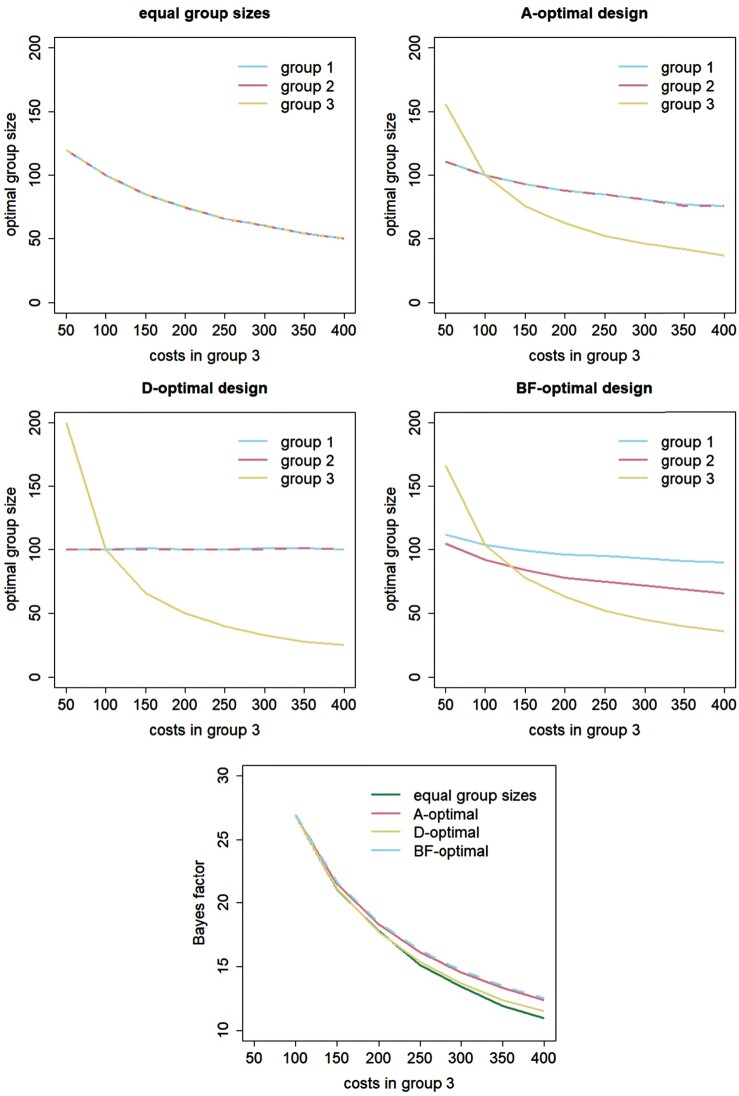
Effect of the costs in group 3 on optimal group sizes and the Bayes factor.

**Figure 2.**
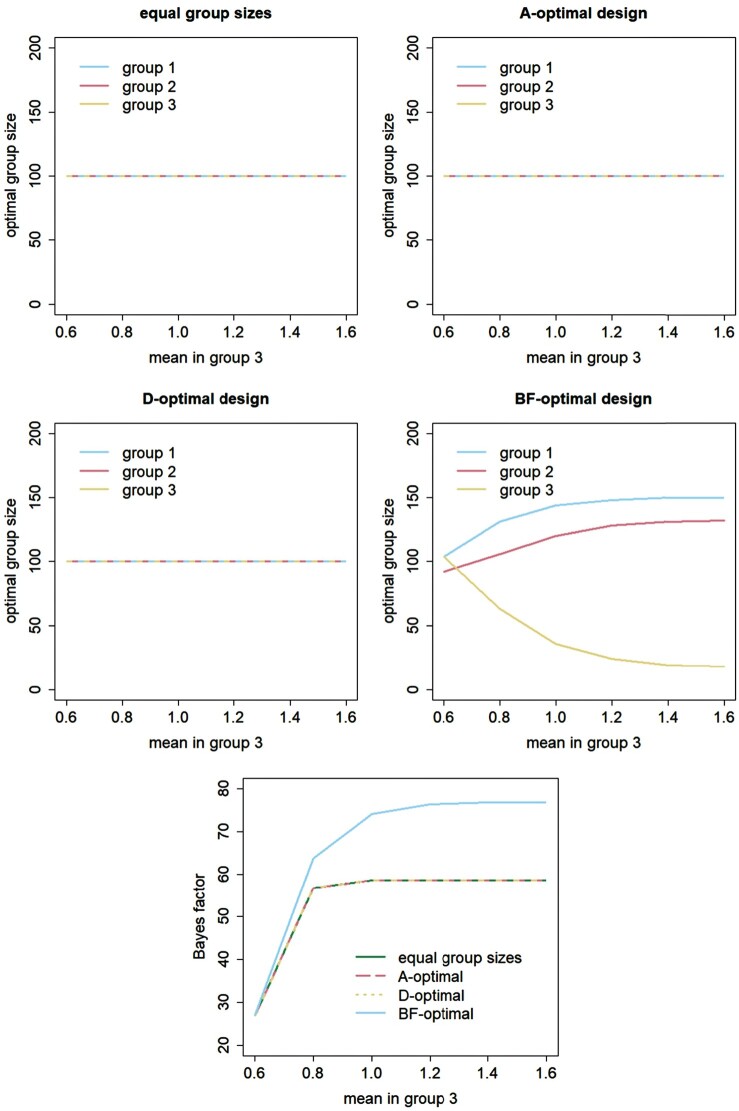
Effect of the mean in group 3 on optimal group sizes and the Bayes factor.

**Figure 3.**
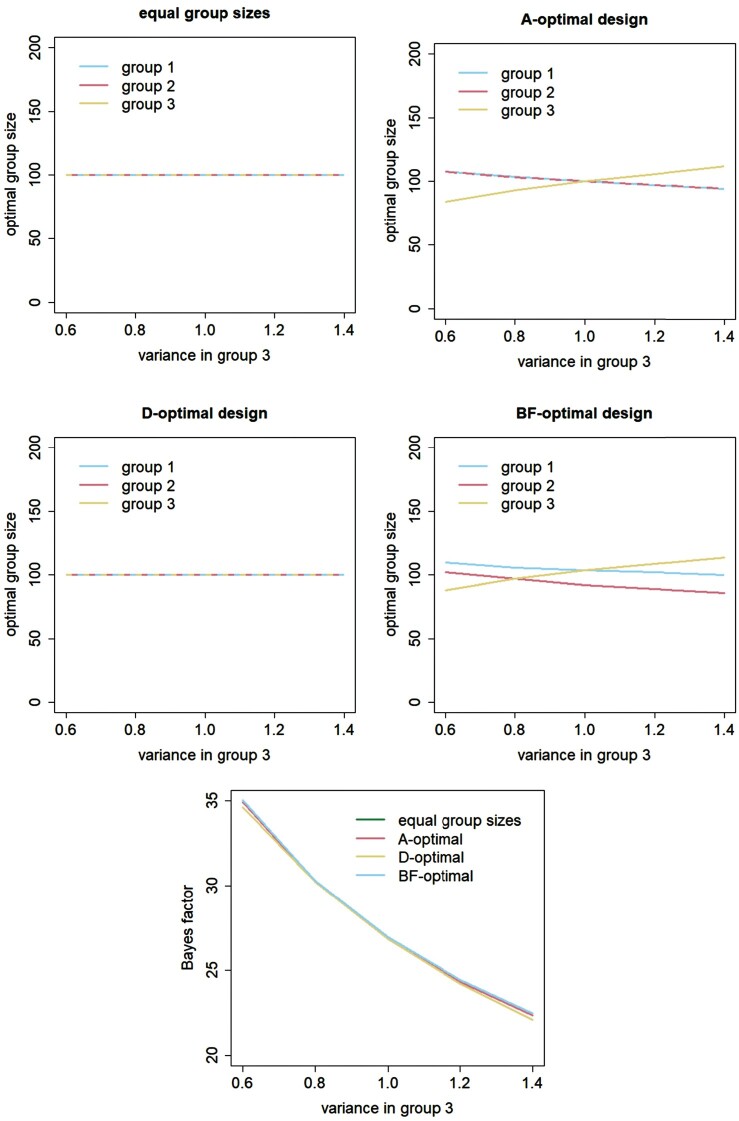
Effect of the variance in group 3 on optimal group sizes and the Bayes factor.

Figures 1–3 explore how the results change when using heterogenous costs and variances and group mean differences of another size. These three factors are considered separately. The first four panels in each of these figures show optimal group sizes for the design with equal group sizes and for the A-optimal, D-optimal and BF-optimal designs, see the main titles of each of these panels. The fifth panel shows the Bayes factors achieved with these four designs.

In Figure 1, the costs in group 3 vary from $c_{3}=50$ till $c_{3}=400$, while keeping the costs in group 1 and 2 constant at $c_{1}=c_{2}=100$. The top left panel shows the relation between $c_{3}$ and the optimal group sizes $n_{1}^{*}$, $n_{2}^{*}$ and $n_{3}^{*}$ for the design with equal group sizes. As is obvious, the common group size decreases when $c_{3}$ increases. The top right panel shows optimal group sizes for the A-optimal design. Only when $c_{3}=100$, the costs in the three groups are equal and hence the three optimal group sizes are equal as well. The group with lowest costs includes the most subjects, meaning that only for $c_{3}<100$ more subjects should be in group 3 than in groups 1 and 2. Groups 1 and 2 have equal costs and hence equal optimal group sizes. The optimal sizes of all three groups become smaller when $c_{3}$ becomes larger, and the decreasing effect of $c_{3}$ on the optimal group size is the strongest for group 3. For the D-optimal design, the three groups should only be of equal size when they have equal costs. Again the design with lowest costs includes the most subjects, so group 3 only has largest optimal size when $c_{3}<100$. Furthermore, $n_{3}^{*}$ declines with increasing $c_{3}$, but $n_{1}^{*}$ and $n_{2}^{*}$ are hardly influenced by $c_{3}$ and equal to each other. All three optimal group sizes for the BF-optimal design decrease with increasing $c_{3}$, and the decreasing effect of $c_{3}$ on the optimal group size is strongest for group 3. Again group 3 is only of largest size when $c_{3}<100$. When $c_{3}\geq 150$, the highest number of subjects should be in group 1, and the lowest number in group 3. The bottom panel shows that the Bayes factors of the four designs decrease with increasing $c_{3}$. This is obvious since as costs of one treatment increase, fewer subjects can be included in the study, which results in a lower Bayes factor. The Bayes factor for the design with equal group sizes is up to 14.4% smaller than that of the BF-optimal design; for the A-optimal design it is at most 1.3% smaller and for the D-optimal design is at most 8.9% smaller.

In Figure 2, the mean outcome in group 3 varies between $\mu_{3}=0.6$ and $\mu_{3}=1.6$, while keeping the means in the other two groups constant at $\mu_{1}=0.2$ and $\mu_{2}=0.4$. The optimal group sizes for the A-optimal and D-optimal design are equal to those for the design that has equal group sizes: $n_{1}^{*}=n_{2}^{*}=n_{3}^{*}=100$. This is obvious since the covariance matrix $\mathrm{cov}(\hat{\mu })$ and the budgetary constraint do no depend on the group means, while the variances and costs do not differ across groups. For the BF-optimal design $n_{3}^{*}$ decreases with increasing $\mu_{3}$. This is obvious, since if $\mu_{3}$ increases, so does the difference in means between groups 2 and 3. Fewer subjects in group 3 are then needed to evaluate the difference in group means between groups 2 and 3 and the budget could better be spend on adding subjects to groups 1 and 2. The optimal sizes of these later two groups indeed increase with increasing $\mu_{3}$. Furthermore, $n_{1}$ is always larger than $n_{2}$. The bottom panel shows that the Bayes factor of all four designs increase with increasing $\mu_{3}$. This is obvious since larger differences between group means are easier to detect and hence result in larger Bayes factors. Furthermore, the BF-optimal design clearly outperforms the other three designs, especially so for large $\mu_{3}$. The design with equal group sizes has the same Bayes factor as the A-optimal and D-optimal design and the value of the Bayes factor does not increase further than 59 when $\mu_{3}$ increases to 1.6.

In Figure 3, the within-group variance $\sigma_{3}^{2}$ in group 3 varies from 0.6 till 1.4, while keeping the other within-group variances constant at $\sigma_{1}^{2}=\sigma_{2}^{2}=1$. Again, the design with equal group sizes incudes 100 subjects per group. Optimal group sizes for the A-optimal design show that $n_{3}^{*}$ increases with increasing $\sigma_{3}^{2}$, and consequently $n_{1}^{*}$ and $n_{2}^{*}$ decrease. The optimal group sizes in the latter two groups are equal. The group with the highest variance thus includes the most subjects. The optimal group sizes for the D-optimal design are equal to each other and do not depend on $\sigma_{3}^{2}$. The optimality criterion to be minimized is the product of the group mean variances and increasing one or more of the within-group variances only results in a multiplication of the value of the optimality criterion, which in its turn does not influence the optimal group sizes. The optimal group sizes for the BF-optimal design show that $n_{3}^{*}$ increases with increasing $\sigma_{3}^{2}$, and consequently $n_{1}^{*}$ and $n_{2}^{*}$ decrease. Group 1 always includes more subjects than group 2. The bottom panel shows that Bayes factors decrease with increasing $\sigma_{3}^{2}$. Larger within-group variances imply that differences in group means are more difficult to detect and hence result in lower Bayes factors. This panel also shows that the Bayes factors obtained with the four designs hardly differ from each other.

The two figures in the supplementary material show that there are also interaction effects for the A- and BF-optimal designs. Figure S1 shows an interaction effect between the variance and costs in group 3 on the optimal group sizes for the A-optimal design. In all three panels, the three group sizes decrease with increasing costs in group 3, but the rate at which they do so depends on the variance in group 3. Figure S2 shows an interaction effect between the variance, the mean and costs in group 3 on the optimal group sizes for the BF-optimal design. It is evident that the relation between the costs in group 3 and optimal group sizes depends on the combination of the mean and variance in group 3. For instance, at low costs in group 3, the optimal group size in group 3 is larger than those in groups 1 and 2 but only when the mean in group 3 is equal to 0.6 (see panels in the left column). In that case, all three optimal group sizes decrease in a monotone fashion as a function of the costs in group 3. However, this is not always the case for larger values of the mean in group 3 (see panels in the middle and right columns).

In Figures 1–3, the effects of $c_{3}$, $\mu_{3}$ and $\sigma_{3}^{2}$ on optimal group sizes and the Bayes factor are evaluated separately while keeping the other parameters constant. In practical settings, the variances as well as the costs may vary across all three groups and also the difference between groups 1 and 2 may be different from the difference between groups 2 and 3. The Shiny app that is available at https://utrecht-university.shinyapps.io/OD_Bayesian_ANOVA/ allows the reader to calculate and compare optimal designs for any combination of the costs, group means and within-group variances, for up to K=6 groups. The time to derive the optimal designs can vary from a few seconds to hours and strongly depends on the number of groups as well as the total sample size, or the budget in relation to the costs. The computation time may be shortened by limiting the ranges of the group sizes, which is done by choosing minimum and maximum group sizes that are not too far apart. Here, practical considerations may play a role. For instance, in one of the conditions, new equipment may be used with which only a limited number of subjects may be treated. As another example, if a treatment may have harmful side effects, then the maximum number of subjects in that condition may be low.

## Illustrative examples

The use of the Shiny app is demonstrated on the basis of two illustrative examples. To use the Shiny app one should first choose how many groups are to be compared by choosing the corresponding tab at the top right. The second step is selecting whether one wants to find the optimal group sizes using a total sample size or a budgetary constraint and then fill out either the total sample size or budget. Next, the means, variances, minimum and maximum group sizes and (in the case of a budgetary constraint) the costs per subject for each of the groups have to be filled out. The group means should be in increasing order, so that group 1 has the lowest mean and group K the highest. The Shiny app then checks if the minimum and maximum group sizes are in accordance with the total sample size or budget so that the design space consists of at least one design. Next, the Shiny app derives the optimal group sizes to test the hypothesis $H:\mu_{1}<\mu_{2}<\ldots <\mu_{K}$ against its complement. The reader is invited to replicate the results below using the Shiny app.

### Multidisciplinary pain management

In a study on multidisciplinary pain management [4], three treatment groups were used: no treatment (NT) from a pain center, cognitive–behavioral treatment plus pharmacotherapy (CBT + PCT), and pharmacotherapy alone (PCT). The outcome variable posttreatment health care visits are used to illustrate the optimal design methodology in this paper. The mean scores on this outcome were ${\bar{x}}_{\mathrm{NT}}=17.3,$ ${\bar{x}}_{\mathrm{CBT}+\mathrm{PCT}}=23.9$, and ${\bar{x}}_{\mathrm{PCT}}=45.9$. The corresponding variances were $\sigma_{\mathrm{NT}}^{2}=7.85^{2}=61.6225$, $\sigma_{\mathrm{CBT}+\mathrm{PCT}}^{2}=19.93^{2}=397.2049$, and $\sigma_{\mathrm{PCT}}^{2}=22.99^{2}=528.5401$, showing heterogeneous within-group variances. These estimates are based on group sizes $n_{\mathrm{NT}}=9$, $n_{\mathrm{CBT}+\mathrm{PCT}}=16$, and $n_{\mathrm{PCT}}=6$, and the total sample size was 31. The standardized differences in means are: Cohen’s d for CBT + PCT versus NT is d=0.4 and Cohen’s d for PCT versus CBT + PCT is d=1.1, indicating a medium and large effect, respectively. If we test the informative hypothesis $H:\mu_{\mathrm{NT}}<\mu_{\mathrm{CBT}+\mathrm{PCT}}<\mu_{\mathrm{PCT}}$, we obtain a Bayes factor of 27.8.

The question is whether this is the best allocation of subjects to the three treatment groups. We use optimal design methodology to determine the optimal group sizes given a fixed total sample size of 31. The optimal designs are summarized in Table 2, and as can be seen, they result in Bayes factors of similar magnitude. The design with group sizes $n_{\mathrm{NT}}^{*}=8$, $n_{\mathrm{CBT}+\mathrm{PCT}}^{*}=13$, $n_{\mathrm{PCT}}^{*}=10$ results in the highest Bayes factor of 29.7, which is 7% higher than the Bayes factor as obtained with the group sizes used by [4] and 8% higher than the BF factor obtained with the design with equal group sizes. The Shiny app also shows four alternative designs that result in high Bayes factors. For each of these, the largest number of subjects is in the condition CBT + PCT, and the Bayes factor is hardly any lower than that of the BF-optimal design.

**Table 2.** Optimal designs for the study on multidisciplinary pain management.

Design	Optimal group sizes $\left(n_{\mathrm{NT}}^{*},n_{\mathrm{CBT}+\mathrm{PCT}}^{*},n_{\mathrm{PCT}}^{*}\right)$	Bayes factor
Cipher *et al.* study	(9, 16, 6)	27.8
Fixed total sample size		
Equal group sizes	(10, 10, 10)	27.4
A-optimal	(5, 12, 14)	27.8
D-optimal	(10, 10, 11)	28.5
BF-optimal	(8, 13, 10)	29.7
Budgetary constraint		
Equal group sizes	(9, 9, 9)	24.6
A-optimal	(5, 12, 9)	24.0
D-optimal	(14, 14, 5)	25.3
BF-optimal	(9, 16, 6)	27.8

One may also calculate the optimal group sizes given a budgetary constraint. The average costs in the study per treatment condition were $c_{\mathrm{NT}}=\$2328.58$, $c_{\mathrm{CBT}+\mathrm{PCT}}=\$2695.12$, and $c_{\mathrm{PCT}}=\$6281.18$, which gives total costs of $n_{\mathrm{NT}}*c_{\mathrm{NT}}+n_{\mathrm{CBT}+\mathrm{PCT}}*c_{\mathrm{CBT}+\mathrm{PCT}}+n_{\mathrm{PCT}}*c_{\mathrm{PCT}}=\$101766.22$. We use these total costs as a budget in the determination of the optimal group sizes. Table 2 shows that the group sizes as used in the study by Cipher result in the highest Bayes factor. This Bayes factor is 13% higher than the one obtained with the design with equal group sizes. The app shows that alternative designs with a high Bayes factor also allocate the highest number of subjects to the condition CBT + PCT. For all designs in Table 2, the Bayes factor shows strong support for the informative hypothesis (see Table 1).

### Psychological status and asthma outcomes

The second example is based on a study on psychological status and asthma outcomes [11], which has been used before [29] to demonstrate methodology to obtain highest power for the test on the interaction effect. We consider the outcome variable emergency service use. The design was 2 × 2 factorial with factors attack context (recent attack versus control) and panic fear (low versus high). So there were four groups in this study: recent attack with low panic fear (AL), recent attack with high panic fear (AH), control with low panic fear (CL), control with high panic fear (CH). The group means were $\mu_{\mathrm{CL}}=0.13$, $\mu_{\mathrm{CH}}=0.38$, $\mu_{\mathrm{AH}}=0.42$ and $\mu_{\mathrm{AL}}=1.23$, the variances were $\sigma_{\mathrm{CL}}^{2}=0.34^{2}=0.1156$, $\sigma_{\mathrm{CH}}^{2}=0.77^{2}=0.5929$, $\sigma_{\mathrm{AH}}^{2}=0.72^{2}=0.5184$ and $\sigma_{\mathrm{AL}}^{2}=0.83^{2}=0.6889$. The group sizes were $n_{\mathrm{CL}}=23$, $n_{\mathrm{CH}}=13$, $n_{\mathrm{AH}}=24$ and $n_{\mathrm{AL}}=13$ (i.e. total sample size of 73). If we test the hypothesis $H:\mu_{\mathrm{CL}}<\mu_{\mathrm{CH}}<\mu_{\mathrm{AH}}<\mu_{\mathrm{AL}}$ using these group sizes, means and variances, then we obtain a Bayes factor of 25.3.

The optimal group sizes given a fixed total sample size of 73 are $n_{\mathrm{CL}}^{*}=20$, $n_{\mathrm{CH}}^{*}=2$, $n_{\mathrm{AH}}^{*}=2$ and $n_{\mathrm{AL}}^{*}=49$. The corresponding Bayes factor is as high as 92.8, which is much higher than the Bayes factor obtained with the other designs (see Table 3). However, with group sizes of just 2, one cannot estimate within-group variances well. Suppose we require group sizes of at least 10. Then the optimal group sizes of the BF-optimal design are $n_{\mathrm{CL}}^{*}=18$, $n_{\mathrm{CH}}^{*}=10$, $n_{\mathrm{AH}}^{*}=10$ and $n_{\mathrm{AL}}^{*}=35$ and the Bayes factor is 43.8, which is 73% higher than the one obtained with the group sizes in the original study.

**Table 3.** Optimal designs for the study on psychological status and asthma outcomes.

Design	Optimal group sizes $\left(n_{\mathrm{CL}}^{*},n_{\mathrm{CH}}^{*},n_{\mathrm{AL}}^{*},n_{\mathrm{AH}}^{*}\right)$	Bayes factor
Greaves *et al.* study	(23, 13, 24, 13)	25.3
Fixed total sample size		
Equal group sizes	(18, 18, 18, 18)	28.2
A-optimal	(10, 21, 20, 22)	24.9
D-optimal	(18, 18, 18, 19)	28.8
BF-optimal	(20, 2, 2, 49)	92.8
Budget constraint		
Equal group sizes	(15, 15, 15, 15)	25.4
A-optimal	(16, 22, 18, 13)	24.3
D-optimal	(50, 22, 22, 8)	25.1
BF-optimal	(28, 3, 2, 23)	56.5

The costs of the four groups were $c_{\mathrm{CL}}=\$82.94$, $c_{\mathrm{CH}}=\$242.44$, $c_{\mathrm{AH}}=\$267.96$ and $c_{\mathrm{AL}}=\$784.94$ [29]. The total costs of the study were thus $21692.00. The budgetary constrained optimal design using these total costs as budget has optimal group sizes $n_{\mathrm{CL}}^{*}=28$, $n_{\mathrm{CH}}^{*}=3$, $n_{\mathrm{AH}}^{*}=2$ and $n_{\mathrm{AL}}^{*}=23$. The corresponding Bayes factor is 56.5 and the amount of money spent is $21639,18, which is slightly lower than the budget. Again, the two middle groups are of very small size. If minimum group sizes of 10 are required, then the optimal group sizes are $n_{\mathrm{CL}}^{*}=39$, $n_{\mathrm{CH}}^{*}=10$, $n_{\mathrm{AH}}^{*}=10$ and $n_{\mathrm{AL}}^{*}=17$. The amount of money spent is $21682.64, again slightly lower than the budget. The Bayes factor is 33.5, which is 32% higher than the one obtained with the group sizes in the original study. The BF-optimal also clearly outperforms the A- and D-optimal designs and the design with equal group sizes in terms of its Bayes factor.

## Discussion and conclusions

This paper developed a methodology and a Shiny app to study optimal group sizes when testing an inequality-constrained informative hypothesis using the Bayes factor and either the total sample size or budget is fixed beforehand. The designs that resulted in the highest Bayes factor were compared to the design with equal group sizes and the A- and D-optimal designs from the framework of null hypothesis significance testing. While the group sizes for the latter three designs only depend on the variances and costs per subject, the group sizes of the BF-optimal design also depend on the differences in group means. For the BF-optimal design, the size of a group decreases if the costs of that group increase, which is obvious since it is then more beneficial to allocate more subjects to the less expensive groups. Furthermore, the size of a group decreases if the difference between its mean and that of other groups increases. This is also obvious since larger differences in group means are easier to detect than smaller differences. Finally, the size of a group increases with the variance in that group. Higher variance implies that the group mean is estimated less efficiently, and hence, a higher group size is required.

The two examples demonstrated the use of the Shiny app. In the first example, the BF-optimal designs hardly outperformed or were equal to the design that was used in the study that motivated this example. In the second example, the BF-optimal clearly outperformed the design that was used in the original study.

To use the methodology in this paper, one should be able to provide a priori estimates of the mean and variance in each of the groups. Such values may come from a pilot study, similar studies in the literature, or an expert’s expectations. Alternatively, one may select ranges of plausible values of the model parameters to perform a sensitivity analysis. Future research may focus on more formal, robust optimal designs, similar to maximin optimal designs in the framework of null hypothesis significance testing [39]. However, it may be very computationally intensive to find such robust optimal designs, especially so when the number of groups is large, and it has to be seen if this is feasible with the computing power of current computers.

The BF-optimal design is sought under a user-specified order of the group means in an inequality-constrained informative hypothesis. If the true order of the group means in the data is different, then the optimal design may perform poorly. Future research may focus on the derivation of the BF-optimal design under the uncertainty of the ordering of group means. Related to that, one may also study BF-optimal designs when multiple competing informative hypotheses are considered. It may then turn out that the design that results in the highest Bayes factor for one hypothesis performs poorly for another hypothesis, and vice versa. This may be resolved by deriving a design that performs well for each hypothesis. Here, there are similarities to a compound optimal design in the framework of null hypothesis significance testing [6].

Another direction for future research is finding the BF-optimal design in the case the informative hypothesis includes equality constraints to reflect that some or all group means are equal to each other. It should then first be studied if, and if yes, to what extent the optimal group sizes depend on the chosen fraction to construct the prior distribution. One may then also study the robustness of the optimal design in the case where such an equality constraint is not present in the data. Future research may also focus on studies with binary outcomes, for instance, studies in (pharmaco-) epidemiology where different medications are compared on patients’ recovery or survival. My first guess is that if the hypothesis is formulated in terms of probabilities, such as $H_{1}:p_{1}<p_{2}<\ldots <p_{K}$, then the optimal design can be found by replacing the means $\mu_{k}$ in the app by the probabilities $p_{k}$ and the variances $\sigma_{k}^{2}$ by $p_{k}(1-p_{k})$**.** When a covariate is thought to have an effect on the outcome, the data should be analyzed by means of a logistic regression model that includes dummy variables to represent the different groups and the covariate. The hypothesis can then be formulated in terms of regression coefficients corresponding to those dummy variables, and future research will focus on the derivation of the optimal design. Other directions of future research are the derivation of BF-optimal designs for studies with co-primary endpoints and for studies that involve nested data, such as cluster randomized trials and multicenter trials [34].

To my knowledge, this is the first paper that studies optimal group sizes in the case of an inequality-constrained informative hypothesis to be tested using the Bayes factor. The BF-optimal design may outperform the design with equal group sizes and designs that are optimal in the framework of null hypothesis significance testing. The Shiny app facilitates the use of the methodology and hence allows the reader to derive the BF-optimal design for his/her study at hand. As such, the use of a study design that performs poorly in terms of the Bayes factor may be avoided.

## Supplementary Material

Supplementary Material.pdf

## Data Availability

Data sharing is not applicable to this article as no datasets were generated or analyzed during the current study.
