# Supplementary material for: Optimal group sizes for testing group mean differences using the Bayes factor
Source: J Appl Stat. 2025 Jul 24;53(4):710–28. doi: 10.1080/02664763.2025.2534898 (PMC12981267; doi:10.1080/02664763.2025.2534898)
Supplement: Supplementary Material.pdf [file CJAS_A_2534898_SM9167.pdf]

Supplementary material.

Optimal group sizes for testing group mean differences using the Bayes factor

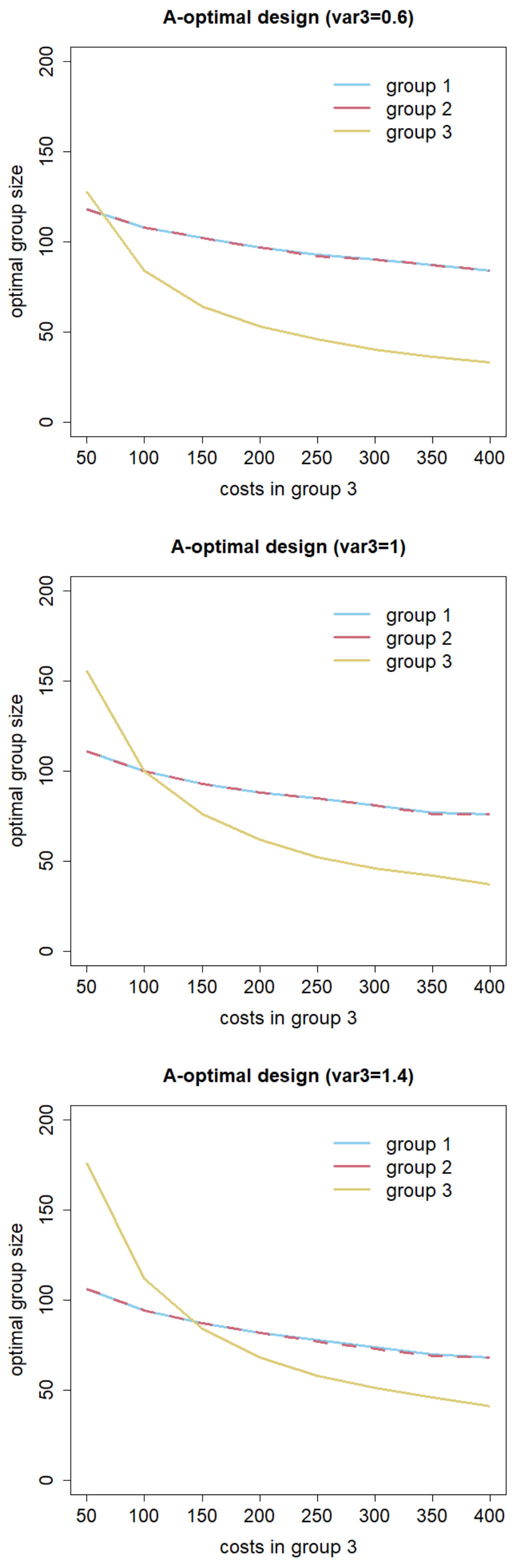

Figure S1. The effect of the costs in group 3 and the variance in group 3 on optimal group sizes for the A-optimal design.

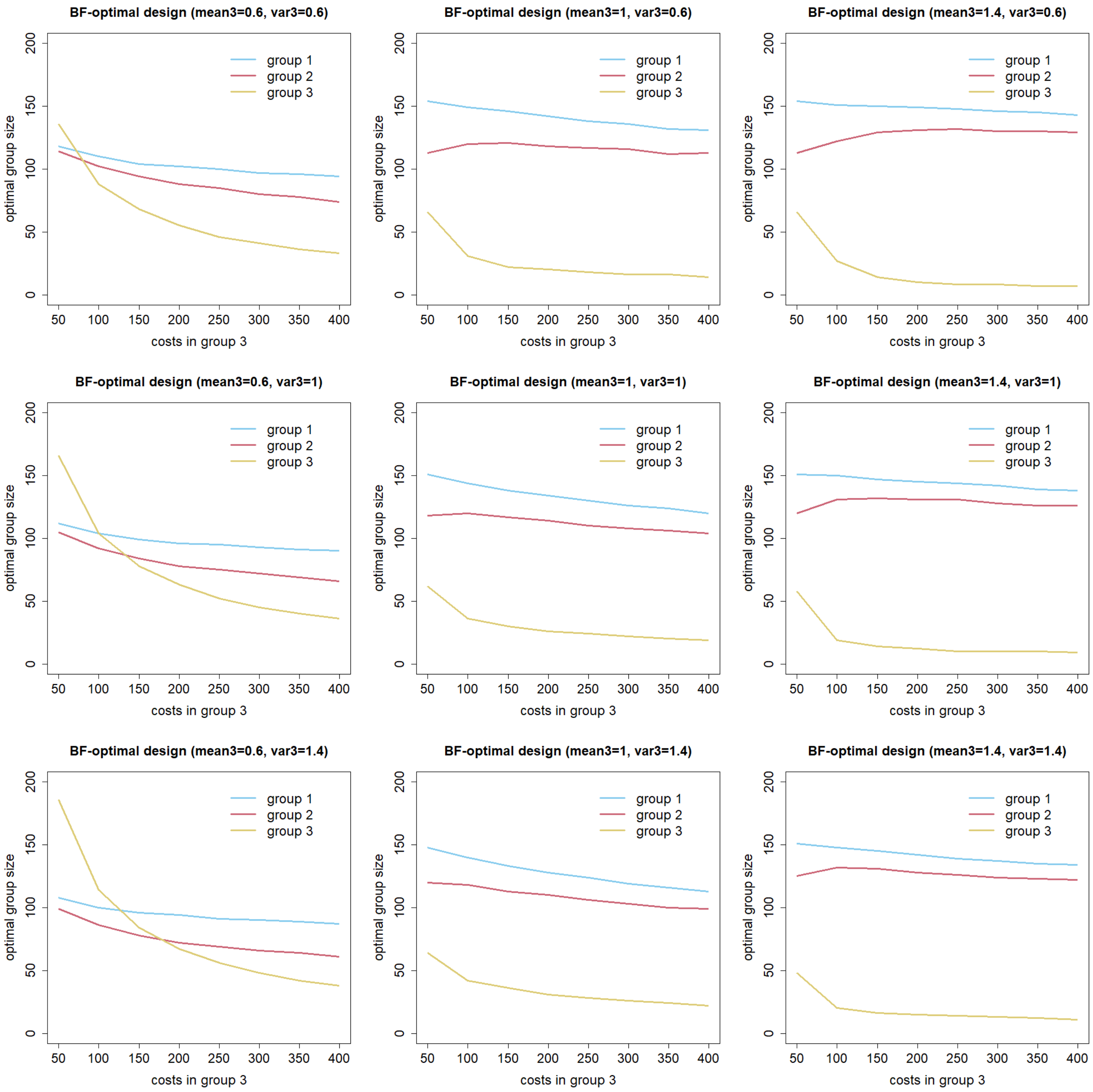

Figure S2. The effect of the costs in group 3, the variance in group 3 and the mean in group 3 on optimal group sizes for the BF-optimal design.
